# Factors Affecting the Academic Achievement of Nursing College Students in a Flipped Learning Simulation Practice

**DOI:** 10.3390/ijerph18115970

**Published:** 2021-06-02

**Authors:** Minkyung Gu, Sohyune Sok

**Affiliations:** 1 Department of Nursing, College of Science and Technology, Daejin University, Pocheon-si 1115, Korea; g-minkyung@hanmail.net; 2 College of Nursing Science, Kyung Hee University, 26, Kyungheedae-ro, Dongdaemun-gu, Seoul 02447, Korea

**Keywords:** simulation, flipped learning, nursing student, academic achievement

## Abstract

A flipped learning simulation practice is composed of two parts. First, it involves learning a practical subject in advance via video in a place other than the classroom, then performing a simulation practice consisting of pre-learning, simulation, and debriefing sections. This study was performed to determine and confirm the factors affecting the level of academic achievement of nursing college students in a flipped learning simulation practice. A cross-sectional descriptive design was used. The participants were 160 nursing students who had experience of a flipped learning simulation practice in a nursing college in South Korea. The factors measured were the general characteristics of the participants, the participants’ academic achievement, the analysis ability of the flipped learning class, the participants’ self-directed learning preparation, the participants’ self-efficacy, and the participants’ learning satisfaction. Data were collected from September to November, 2019. As a result of the analysis, we found that the factor that had the greatest influence on the academic achievement of nursing students was self-efficacy, followed by gender, flipped learning education experience, learning satisfaction, age, and the analysis ability of the flipped learning class. In the nursing practicum, nursing educators need to pay attention to the use of flipped learning simulation practice and the factors affecting the academic achievement of nursing college students. In flipped learning simulation practice, specific interventions and strategies are required to improve the academic achievement of nursing college students.

## 1. Introduction

In recent decades, university education has been rapidly changing from instructor-centered education to learner-centered education, and teaching-learning methods are being actively enhanced to respect and satisfy learners’ educational needs [1,2,3]. Accordingly, nursing education also requires a teaching-learning method that will enable nursing students to actively seek out the various nursing problems that arise in actual clinical practice. This teaching-learning method should be based on nursing student-centered participation, induction, and motivation, and it will help nursing students prepare for self-directed learning [4].

The teaching-learning method called flipped learning is an inverse-concept learning approach that breaks away from traditional lecture-based education methods [5]. Instead, the learner learns in advance in a place other than the classroom, using video lessons prepared by the instructor that utilize the concepts that the learner acquires in their regular school class [3,6]. In other words, flipped learning is a method in which the learner participates in class after engaging in self-directed learning and after acquiring prior knowledge [6]. Flipped learning has started to draw attention of late, largely because it has high computer and mobile accessibility and ease of use and because learners can broaden their knowledge through reasoning and participate in activity-oriented classes [3,5]. In particular, it has the advantage of providing sufficient orientation to decrease the tension, anxiety, and stress of nursing students, which is a limitation of simulation practicum in the nursing colleges in South Korea [2,3]. Thus, it can reduce nursing students’ simulation practicum participation avoidance [7,8]. Moreover, practical practicum training using flipped learning can be designed by the students themselves, thereby increasing their self-efficacy and learning satisfaction [1,8]. Above all, it can have continuous effects on nursing students’ academic achievement.

The analysis and evaluation of a flipped learning simulation practice should be conducted in order to improve the quality of nursing education, ensuring that it enables nursing students to acquire professional and vocational skills rather than evaluating the results of nursing education based only on the students’ academic achievement, as occurs in the existing nursing education system [8,9]. Flipped learning simulation practice is a teaching-learning method in which a learner first takes a practical class via video in a place other than the classroom and then performs a simulation practice consisting of pre-learning, simulation, and debriefing sections [6,8]. Flipped learning simulation practice reduces learners’ fear of the tasks they will be perform by allowing them to complete their knowledge acquisition through mutual cooperative learning with the instructor and making them explore problems and their respective solutions using a combination of the knowledge, skills, and attitudes they have gained [1,9]. In addition, while experiencing practical practicum using flipped learning, students can generate self-directed feedback while conducting actual nursing activities, establish nursing plans that directly solve problems, and simultaneously feel a sense of accomplishment in their nursing practice [3,9]. Flipped learning practical tasks can also be effective for the continuous learning satisfaction of nursing students [9].

Nevertheless, studies have yet to be conducted to determine the factors affecting the academic achievement of nursing college students utilizing a flipped learning simulation practice, such as those students in nursing colleges in South Korea. Therefore, this research attempts to explore the experience of a flipped learning simulation practice in a comprehensive and in-depth manner, and to determine the nursing college students’ academic achievement in a flipped learning simulation practice. This study also attempts to investigate students’ ability to analyze a flipped learning class, and students’ self-directed learning preparation, self-efficacy, and learning satisfaction, which were derived from the previous research results as predictor variables [1,8,9,10]. This study intends to provide basic data for the development of a simulation training scenario program using efficient flipped learning. It is hoped that this will further help nursing students to improve their academic achievement in practical practicum; enhance their ability to carry out clinical practice; and ultimately nurture competent, expert nurses. The aim of the study is to examine the factors that affect the level of academic achievement among nursing college students in a flipped learning simulation practice.

## 2. Materials and Methods

### 2.1. Study Design and Participants

A cross-sectional descriptive design was used. The participants were 160 nursing students who had experience of a flipped learning simulation practice in a nursing college in South Korea. Flipped learning simulation practice is a teaching-learning method used in a nursing practicum for nursing college students. Flipped learning simulation practice is composed of two parts, including learning a practical subject in advance via video in a place other than the classroom and then performing a simulation practice consisting of pre-learning, simulation, and debriefing sections. Eligibility criteria were that participants were 20 years of age or older, consented to participate in this study, understood the purpose of this study, had completed the fundamental nursing course in the second year of the nursing curriculum, and had no clinical nursing practice experience. Of the 165 questionnaires, 161 (97.6%) were answered. Only 160 questionnaire responses were included in the final data set due to incomplete data. For sample size suitability (*n* = 146), the G power 3 analysis software was used, which included 146 samples. This was calculated on the basis of a significance alpha = 0.05, effect size = 0.15, and power = 0.95 [11]. Therefore, the sample size used in the study was feasible.

### 2.2. Measures

The survey of the general characteristics of study participants was created by researchers and consisted of five items, including gender, age, religion, living with others, and satisfaction with the nursing major. 

A Korean version of the academic achievement scale designed by Rovai et al. [12] was created by Park et al. [13]. This was utilized to examine the students’ levels of academic achievement, and involved a total of nine questions with a 5-point Likert scale. The scores ranged from 9 to 45 points. The higher a respondent’s score, the higher their level of academic achievement. In the study by Rovai et al. [12], Cronbach’s α = 0.79, and reliabilities in this study were Cronbach’s α = 0.87.

Leem and Kim developed a scale to measure a student’s ability to analyze the flipped learning class for Korean nursing college students and their teacher [14]. This scale was utilized to examine the level of analytical ability of the flipped learning class. It was created using a total of 20 questions with a 5-point Likert scale, and the score ranged from 20 to 100 points. The higher a respondent’s score, the higher the level of analysis ability of the flipped learning class. In the study by Leem and Kim [14], Cronbach’s α = 0.83, and reliability in this study was Cronbach’s α = 0.79.

The self-directed learning preparation scale designed by Guglielmino [15] was adapted by Cho and Roh [16] for Korean nursing students. This scale was utilized to examine students’ levels of self-directed learning preparation. It was created using a total of 24 questions with a 5-point Likert scale, and the score ranged from 24 to 120 points. The higher a respondent’s score, the higher their level of self-directed learning preparation. In the study by Guglielmino [15], Cronbach’s α = 0.94, and reliability in this study was Cronbach’s α = 0.94.

The self-efficacy scale designed by Sherer et al. [17] was adapted for Korean participants by Hong [18]. This was used to examine the students’ levels of self-efficacy. This scale included questions regarding general self-efficacy (17 items) and social self-efficacy (6 items). It was comprised of a total of 23 questions with a 5-point Likert scale, and the score ranged from 23 to 115 points. The higher a respondent’s score, the higher their level of self-efficacy. In Hong’s study [18], Cronbach’s α = 0.86, and reliability in this study was Cronbach’s α = 0.88.

The learning satisfaction scale was designed by You and You [19]. This was utilized to examine the students’ levels of learning satisfaction. It was created using a total of 24 questions with a 5-point Likert scale, and the score ranged from 24 to 120 points. The higher a respondent’s score, the higher their level of learning satisfaction. In the original paper by You and You [19], Cronbach’s α = 0.94, and the reliability of the scale in this study was Cronbach’s α = 0.97.

### 2.3. Procedures 

Data were collected from September to November, 2019. A researcher visited the college of nursing to obtain permission. A flipped learning simulation practice is composed of two parts, including teaching a practical class in advance via a video in a place other than the classroom and then performing a simulation practice consisting of pre-learning, simulation, and debriefing sections. The nursing college students who experienced the flipped learning simulation practice as described above were the preliminary participants of this study. Researchers contacted the preliminary nursing college student participants and described the aims of this study as well as the details of participation and the questionnaire to be utilized. Researchers received written informed consent from the nursing college students who consented to participate in this study. The questionnaire was given only to nursing college students who consented to participate in the study. Afterwards, the completed questionnaires were collected. The questionnaire involved self-reporting and was kept by the researchers. Each subject took about 20–25 min to complete the questionnaire. 

### 2.4. Statistical Analysis 

Data were analyzed by SPSS Statistics, PC+ version 23.0 (IBM, Armonk, NY, USA). The general characteristics of the study participants and the study variables were analyzed using descriptive statistics. Differences in academic achievement according to the general characteristics of the participants were analyzed using a *t*-test, and an ANOVA was conducted with the Scheffe post hoc test. Pearson’s correlation coefficient was utilized to analyze the correlations between academic achievement and related factors. To determine the factors affecting each student’s level of academic achievement, a hierarchical stepwise multiple regression analysis was used. 

### 2.5. Ethical Considerations 

For ethical considerations, this study was approved by the Institutional Review Board of D University in Korea (IRB No. 1040656-201907-SB-01-04). Participants voluntarily participated in this study and were informed that they could withdraw from the study at any time. Researchers also made the participants aware of the confidentiality of the data. Researchers obtained written consent from the study participants.

## 3. Results

### 3.1. General Characteristics of the Study Participants 

The majority of participants were women (women: 76.9%, male: 23.1%). The mean age was 23.34 years old, and 54.4% of participants had no religion. In regards to participants living with others, 71.3% of participants were living with their families. With regard to students’ level of satisfaction with their nursing major, “Moderately satisfied” or “Satisfied” was the most common answer (93.1%) (Table 1).

### 3.2. Levels of Academic Achievement, Analysis Ability of Flipped Learning Class, Self-Directed Learning Preparation, Self-Efficacy, and Learning Satisfaction 

The average of the participants’ academic achievement was 30.63 points, which was higher than the median (27 points). The average analysis ability of the flipped learning class was 61.81 points, which was slightly higher than the median (60 points). The average of the participants’ self-directed learning preparation was 81.70 points, which was higher than the median (72 points). The average of the participants’ self-efficacy was 76.39 points, which was higher than the median (69 points). The average of the participants’ learning satisfaction was 96.18 points, which was higher than the median (72 points) (Table 2).

### 3.3. Differences in Academic Achievement According to the General Characteristics of the Participants

There were differences in the average scores for academic achievement in some participant characteristics related to gender (t = 12.65, *p* < 0.001), age (F = 5.05, *p* < 0.001), and satisfaction with the nursing major (F = 5.70, *p* = 0.004). The results show higher levels of academic achievement in nursing students who were female, who were 22 years old or younger, and who had higher levels of satisfaction with their nursing major (Table 3).

### 3.4. Correlations in Academic Achievement, Analysis Ability of the Flipped Learning Class, Self-Directed Learning Preparation, Self-Efficacy, and Learning Satisfaction

Academic achievement had a significant, positive relationship with the analysis ability of the flipped learning class (γ = 0.49), self-directed learning preparation (γ = 0.46), self-efficacy (γ = 0.63), and learning satisfaction (γ = 0.49) (Table 4). 

### 3.5. Factors Affecting Academic Achievement

We tested the assumptions of the regression model to see if this study was appropriate for regression analysis. Through the examination of the residual plot, homoscedasticity was confirmed. In order to test for autocorrelation, which may result in errors, the Durbin–Watson statistic was used to check the independence of the residuals. The statistic was 1.60, which was between 1.59 and 1.76, thereby satisfying the assumptions of a regression equation without autocorrelation. In addition, the tolerance of multicollinearity was 0.29–0.97, which was more than 0.10, and the variance inflation factor (VIF) was 1.03–3.49, which was not more than 10, showing that none of the variables had a multicollinearity problem.

A hierarchical stepwise multiple regression analysis was attempted in order to analyze the factors affecting the level of academic achievement in a flipped learning simulation practice for nursing college students. For the analysis results, the Step 1 regression model (with the general characteristics of nursing college students) was statistically significant. The statistically significant variables in Step 1 were gender, age, and satisfaction with the nursing major, and the explanatory power of the Step 1 regression model was 20.6%. In Step 2 of this study, analyses of students’ flipped learning education experience and internet access were added. In this stage, the general characteristics of nursing college students, flipped learning education experience, and internet access were inputted, and the statistically significant variables were found to be gender, age, levels of satisfaction with the nursing major, and flipped learning education experience. The Step 2 regression model was statistically significant, and the explanatory power was 24.0%, up 3.4% from Step 1. In Step 3, the students’ ability to analyze the flipped learning class, self-directed learning preparation, self-efficacy, and learning satisfaction (which were the main study variables) were additionally inputted. The statistically significant variables in this stage, in which the general characteristics of nursing students and the main study variables were inputted, were gender, age, flipped learning education experience, analysis ability of the flipped learning class, self-efficacy, and learning satisfaction. The Step 3 regression model was also significant, and its explanatory power was 54.1%, which was an increase of 30.1% compared to that of the Step 2 regression model.

In this study, the nursing college students’ academic achievement in a flipped learning simulation practice was influenced the most by self-efficacy (β = 0.41, *p* < 0.001), followed by gender (β = −0.26, *p* = 0.010), flipped learning education experience (β = 0.24, *p* < 0.001), learning satisfaction (β = 0.22, *p* < 0.002), age (β = −0.21, *p* < 0.024), and analysis ability of the flipped learning class (β = 0.16, *p* < 0.036) (Table 5).

## 4. Discussion

This study attempted to determine the academic achievement of nursing college students being taught via a flipped learning simulation practice, and to identify the predictor variables, including analysis ability of the flipped learning class, self-directed learning preparation, self-efficacy, and learning satisfaction, in order to work towards an intervention plan for a simulation practice using systematic flipped learning.

In this study, it was seen that the academic achievement of nursing students being taught via a flipped learning simulation practice was relatively higher than the average. This supports the study finding of You and Kim [20] that the academic achievement of nursing college students improved, and they noticed problems with situations faster, when they did prior learning by applying real clinical cases using flipped learning. Additionally, a study by Lewis et al. [21] and a study by Simpson and Richards [22] support the finding that academic achievement can be increased by promoting the prior learning linked to flipped learning content. The development of a systematic scenario program for a flipped learning simulation practice that can motivate nursing students to obtain self-directed prior learning, and the role of the instructor, are both considered very important [23].

In this study, the female students showed higher academic achievement than the male students. This supports the findings of Kang [24] and of Kim and Kim [25]. In these studies, the female students’ academic achievement was superior because their ability to manage assessment items, including the usual assignments, was superior when compared to that of the male students. However, as this study was conducted for nursing colleges, where the majority of students are female, it is necessary to reconfirm the factors affecting a flipped learning simulation practice. This could be done in the future with by ensuring the homogeneity of participants, and by repeating and expanding the research across various courses.

This study showed that the younger the nursing students and the higher the degree of their satisfaction with the nursing major, the higher their academic achievement. It is thought that the younger a nursing student is, the higher his or her curiosity about the subject, as well as his or her interest in mutually cooperative learning, which allows a student to learn along with their fellow students [26,27]. Moreover, in this study, the various core teaching multimedia materials devised by the instructor managed to arouse the interest of the nursing college students in the flipped learning simulation practice [5,26]. Therefore, educational programs are needed that are able to maximize experiential learning, which can increase the interest of nursing college students by applying various innovative teaching-learning methods and promoting teamwork activities.

The results from this study show that the academic achievement of nursing college students has significant positive correlations with the analysis ability of the flipped learning class, self-directed learning preparation, self-efficacy, and learning satisfaction. This supports the findings of Park et al. [13] and Cho and Kim [23] that the higher the academic achievement of a nursing student is, the more they will actively lead their own learning, which increases their self-efficacy and positively affects their learning satisfaction [28]. Therefore, instructors need to use a variety of teaching-learning methods and provide flexible learning environments that can increase the self-efficacy and learning satisfaction of their nursing college students, while reviewing their understanding of nursing college students ability to analyze the flipped learning [5]. Above all, instructors should design their classes for simulation practicum using specific and systematic flipped learning [29,30].

In this study, self-efficacy was found to be the most important variable affecting the nursing college students’ flipped learning simulation practice, followed by gender, flipped learning education experience, learning satisfaction, age, and the analysis ability of the flipped learning class. These results support the finding of Cho and Kim’s study [23] that nursing college students’ preparation for self-directed prior learning in a health assessment practical task using flipped learning was also effective in improving the students’ self-efficacy and learning satisfaction, and the finding that university students’ self-efficacy and learning satisfaction increased when a strategic flipped learning teaching-learning method was used [10,28]. The instructor running the simulation practicum should arrange for the collaborative learning of nursing students in order to achieve repetitive prior-learning linkage, and should aim to increase their self-efficacy by holding advance group discussions on the details of the collaborative learning process through flipped learning and allow them to perform the analysis of the flipped learning class in various courses [28,31]. In addition, it is necessary to increase the nursing college students’ self-learning time by providing them with the opportunity to learn repeatedly through the flipped learning method [3,7,32], and above all, to broaden their educational experience of flipped learning.

The results from this study are significant in that they can be utilized as basic data for the creation of a simulation practice intervention program using flipped learning for nursing college students.

As for the limitations of the study, care should be taken to expand the findings of this study and to explain the factors affecting the academic achievement of all nursing college students in flipped learning simulation training. This is especially important, as there have been almost no previous studies on flipped learning simulation training for nursing college students.

## 5. Conclusions

In conclusion, the most influential factors affecting the academic achievement of nursing college students were self-efficacy, gender, flipped learning education experience, learning satisfaction, age, and the analysis ability of the flipped learning class, in order.

In order to improve the academic achievement of nursing college students, simulation training using flipped learning and systematic video lecture content for nursing college students should be provided for repeated prior learning. Nursing college students’ self-efficacy and academic achievement should be increased through preparation for self-directed learning by increasing their educational experience of flipped learning. Moreover, the instructors running the simulation training using flipped learning for nursing college students need to stimulate the interest of their students and increase their learning satisfaction by arranging specific procedures and strategies for flipped learning class analysis, encouraging students to apply this learning to various clinical cases. In particular, it is necessary to develop a systematic nursing flipped learning simulation training model that takes into consideration the students’ age and school year so that sufficient flipped learning training experience can be obtained.

In the future, it is necessary to develop a simulation scenario program that utilizes professional and highly skilled flipped learning. In order to develop this program, it is necessary to gain a wider understanding of the nature of effective flipped learning simulation training and to conduct experimental research to verify the efficacy of the program.

## Figures and Tables

**Table 1:** General characteristics of study participants.

Characteristics	*n*	%
Gender:		
Male	37	23.1
Female	123	76.9
Age (years):		
≤22	68	42.6
23	38	23.8
24	21	13.2
≥25	33	20.4
Religion:		
Religious	73	45.6
Non-religious	87	54.4
Living with others:		
With family	114	71.3
Alone (Dormitory)	40	25.0
Other	6	3.7
Satisfaction with the nursing major:		
Satisfied	74	46.3
Moderately satisfied	75	46.8
Dissatisfied	11	6.9

**Table 2:** Levels of academic achievement, analysis ability of the flipped learning class, self-directed learning preparation, self-efficacy, and learning satisfaction.

Variables	Mean ± SD	Min	Max	Range Point
Academic achievement	30.63 ± 5.84	19.00	45.00	9–45
Analysis ability of the flipped learning class	61.81 ± 12.16	39.00	92.00	20–100
Self-directed learning preparation	81.70 ± 15.93	40.00	120.00	24–120
Self-efficacy	76.39 ± 11.51	56.00	104.00	23–115
Learning satisfaction	96.18 ± 18.52	50.00	120.00	24–120

**Table 3:** Differences in academic achievement according to the general characteristics of the participants.

Characteristics	Mean ± SD	t or F	*p*	Scheffe
Gender:		12.65	<0.001 *	
Male	29.76 ± 5.29			
Female	33.51 ± 6.67			
Age (years):		5.05	<0.001 *	a > b,c,d
≤22	32.34 ± 2.55 ^a^			
23	31.13 ± 5.27 ^b^			
24	28.76 ± 6.14 ^c^			
≥25	29.12 ± 5.85 ^d^			
Religion:		0.16	0.692	
Religious	30.42 ± 5.43			
Non-religious	30.79 ± 6.19			
Living with others:		2.87	0.060	
With family	30.20 ± 6.32			
Alone (Dormitory)	32.30 ± 4.26			
Others	27.50 ± 1.09			
Satisfaction with the nursing major:		5.70	0.004 *	a > b,c
Satisfied	32.26 ± 4.94 ^a^			
Moderately satisfied	29.23 ± 6.36 ^b^			
Dissatisfied	29.18 ± 5.33 ^c^			

* *p* < 0.05; ^a, b, c, d^ Scheffe post hoc test.

**Table 4:** Correlations in academic achievement, analysis ability of the flipped learning class, self-directed learning preparation, self-efficacy, and learning satisfaction.

Variables	Academic Achievement	Analysis Ability of Flipped Learning Class	Self-Directed Learning Preparation	Self-Efficacy	Learning Satisfaction
Academic achievement	1				
Analysis ability of the flipped learning class	0.49 *	1			
Self-directed learning preparation	0.46 *	0.65 *	1		
Self-efficacy	0.63 *	0.43 *	0.42 *	1	
Learning satisfaction	0.49 *	0.30 *	0.36 *	0.48 *	1

* *p* < 0.05.

**Table 5:** Factors affecting academic achievement.

**Variables**	**Step 1**						
	**B**	**S.E**	**β**	**t**	***p***	**95% CI**	
						**Lower**	**Upper**
Gender	−8.78	1.54	−0.64	−5.68	<0.001 *	−11.83	−5.73
Age	−1.68	0.44	−0.42	−3.83	<0.001 *	−2.54	−0.81
Religion	−0.42	0.85	−0.04	−0.49	0.626	−2.09	1.26
Living with others	0.68	0.77	0.06	0.89	0.376	−0.84	2.20
Satisfaction with the nursing major	−3.02	0.70	−0.32	−4.32	<0.001 *	−4.40	−1.64
Adj R^2^ = 0.206, F = 9.24, *p* < 0.001 *							
**Variables**	**Step 2**						
	**B**	**S.E**	**β**	**t**	***p***	**95% CI**	
						**Lower**	**Upper**
Gender	−8.89	1.52	−0.64	−5.85	<0.001 *	−11.90	−5.90
Age	−1.76	0.43	−0.44	−4.09	<0.001 *	−2.60	−0.91
Religion	−0.92	0.86	−0.08	−1.08	0.281	−2.61	0.76
Living with others	0.43	0.76	0.04	0.56	0.575	−1.08	1.93
Satisfaction with the nursing major	−2.57	0.71	−0.27	−3.63	<0.001 *	−4.00	−1.17
Experience in flipped learning education	3.71	1.25	0.22	2.97	0.003 *	1.24	6.18
Computer and internet accessibility	0.95	1.29	0.05	0.74	0.461	−1.69	3.50
Adj R^2^ = 0.240, F = 8.19, *p* < 0.001 *							
**Variables**	**Step 3**						
	**B**	**S.E**	**β**	**t**	***p***	**95% CI**	
						**Lower**	**Upper**
Gender	−3.61	1.38	−0.26	−2.61	0.010 *	−6.35	−0.88
Age	−0.84	0.37	−0.21	−2.28	0.024 *	−1.57	−0.11
Religion	−0.67	0.68	−0.06	−0.98	0.330	−2.01	0.68
Living with others	0.01	0.62	0.00	0.01	0.999	−1.22	1.22
Satisfaction with the nursing major	0.43	0.63	0.05	0.68	0.499	−0.81	1.67
Experience in flipped learning education	4.20	0.98	0.24	4.29	<0.001 *	2.26	6.13
Computer and internet accessibility	1.46	1.01	0.08	1.45	0.149	−0.53	3.45
Analysis ability of flipped learning class	0.08	0.04	0.16	2.12	0.036 *	0.01	0.15
Self-directed learning preparation	0.01	0.03	0.04	0.47	0.638	−0.04	0.07
Self-efficacy	0.22	0.04	0.41	5.93	<0.001 *	0.14	0.29
Learning satisfaction	0.07	0.02	0.22	3.10	0.002 *	0.02	0.11
Adj R^2^ = 0.541, F = 18.06, *p* < 0.001 *							

Adj. R^2^ = Adjust R-squared; CI = Confidence Interval; * *p* < 0.05.

## Data Availability

No new data were created or analyzed in this study. Data sharing is not applicable to this article.
